# Feasibility of laparoscopic enucleation for hemangioma in special hepatic segments

**DOI:** 10.3389/fsurg.2022.1111307

**Published:** 2023-01-17

**Authors:** Huixing Li, Xuhong Duan, Zhenyu Wu, Yugang Qin

**Affiliations:** ^1^ Department of Hepatobiliary Surgery, Aerospace Center Hospital, Beijing, China; ^2^ Department of Gastroenterology, First Medical Center of Chinese PLA General Hospital, Beijing, China

**Keywords:** hepatic hemangioma, laparoscopy, special location, surgery, prognosis

## Abstract

**Background and aim:**

This study aims to evaluate the safety and efficacy of laparoscopic enucleation for liver hemangioma in special hepatic segments.

**Methods:**

We retrospectively reviewed 58 patients who underwent laparoscopic surgery for hepatic hemangioma at a single center from January 2016 to January 2022. Segments I, IVa, VII, and VIII are defined as special hepatic segments, attributing to the bad visualization and adjacent to important vessels such as hepatic veins and inferior vena cava that lead to a high risk in laparoscopic surgery. Patients were categorized into a special location group (SLG) and a normal location group (NLG) according to the location of hemangioma. General data, intraoperative and postoperative outcomes, and postoperative complications of the two groups were compared and analyzed.

**Results:**

There were no significant differences in age (*p* = 0.288), gender (*p* = 0.331), body mass index (*p* = 0.168), the maximum diameter of hemangioma (*p* = 0.330), ASA risk grading (*p* = 0.615), and comorbidities (*p* > 0.05) between the two groups. The operation time (*p* < 0.001), intraoperative blood loss (*p* < 0.001), and intraoperative blood transfusion rate (*p* = 0.047) were significantly higher in the SLG. The rate of conversion to laparotomy was higher in the SLG, but there was no significant difference (*p* = 0.089). In addition, the exhaust time (*p* = 0.03) and postoperative hospital stay (*p* < 0.01) were significantly shorter in the NLG. The postoperative complications were comparable between the two groups, and there were no perioperative deaths.

**Conclusion:**

Laparoscopic enucleation of hemangioma in special hepatic segments is difficult and has a critical risk of massive bleeding during surgery. Meanwhile, it is also safe, feasible, and effective.

## 1. Introduction

A hepatic hemangioma (HH) is one of the most common benign liver tumors, with incidence ranging from 0.4% to 20% in the general population (1, 2). It develops slowly and has a good prognosis. As for its pathogenesis, some scholars believe that a large number of vascular cells proliferate in the liver during embryonic development, forming a malformed vascular mass that lacks smooth muscle tissue. Furthermore, the changes in the level of acquired endocrine hormones significantly promote its growth (3). Relevant studies suggest that estrogen receptors exist in hepatic hemangioma tissues, and the occurrence and development of hemangiomas are estrogen-dependent. Therefore, the increase in estrogen level caused by female puberty, oral contraceptives, or pregnancy can accelerate the growth of hepatic hemangiomas (4–6). Hepatic hemangiomas can be divided into four histological types, including cavernous hemangioma, capillary hemangioma, sclerosing hemangioma, and hemangioendothelioma, according to the amount of fibrous tissue contained in hemangiomas. A cavernous hemangioma is the most common in clinics (7).

HH is usually asymptomatic and is discovered incidentally by abdominal imaging during routine physical examination. Traditionally, asymptomatic hepatic hemangiomas require no further clinical intervention (8). However, if there is a rapid growth of hemangioma or obvious clinical symptoms such as abdominal pain, compression of adjacent organs, intratumoral bleeding, acute abdomen due to tumor rupture, and Kasabach–Merritt syndrome during the long-term follow-up, further treatment is needed (9–11). Treatment options for HHs are corticosteroid treatment, transcatheter arterial embolization, radiofrequency ablation, surgical resection, and, occasionally, transplantation (12–15). Surgical resection, including segmental hepatectomy and hemangioma enucleation, is the most effective and widely accepted treatment for these symptomatic patients (16, 17). Also, many surgeons favor enucleation for HHs as it is a safe and quick technique with a maximum amount of normal liver parenchyma preserving, low blood loss, and low morbidity and mortality rates for hepatic hemangiomas.

Over the past few decades, with the rapid development of minimally invasive surgery, the continuous innovation of laparoscopic-related instruments and the improvement in endoscopic operation technology, the laparoscopic liver approach has gained widespread acceptance and become the first choice for the treatment of HH. Its safety, efficacy, and advantages have been effectively verified in clinical practice (18–20). However, it still has a high difficulty and risk in laparoscopic surgery for hepatic hemangiomas in special location (Couinaud liver segments I, IVa, VII, and VIII) (21–26), as visualization is the key point in laparoscopic surgery. When HHs are located in these special segments, visualization is limited and it is difficult to reach. Meanwhile, the tumor body is usually adjacent to vessels such as hepatic veins and inferior vena cava and compresses these main vessels. Especially the HH located in segment I has several thin hepatic veins draining directly into the inferior vena cava and is close to the liver hilum. These factors increase the risk of uncontrolled bleeding, which is a threat to patient's life and requires a shift to laparotomy. The aim of this study was to evaluate the safety and efficacy of laparoscopic enucleation for liver hemangiomas in special hepatic segments.

## 2. Materials and methods

### 2.1. Patients

The clinical and postoperative data of 58 patients who underwent laparoscopic surgery for hepatic hemangiomas in the Hepatobiliary Surgery Department of Aerospace Center Hospital from January 2016 to January 2022 were retrospectively reviewed in this study. All patients were accompanied by clinical symptoms. They were diagnosed primarily by enhanced computerized tomography (CT), enhanced abdominal ultrasonography, and/or enhanced magnetic resonance imaging (MRI). All patients' postoperative pathological diagnoses were hepatic cavernous hemangiomas. Written informed consent was obtained from all patients and their authorizers before the surgery. The study was approved by the Committee of Ethics in Aerospace Center Hospital.

### 2.2. Preoperative evaluation

**Figure 1:**
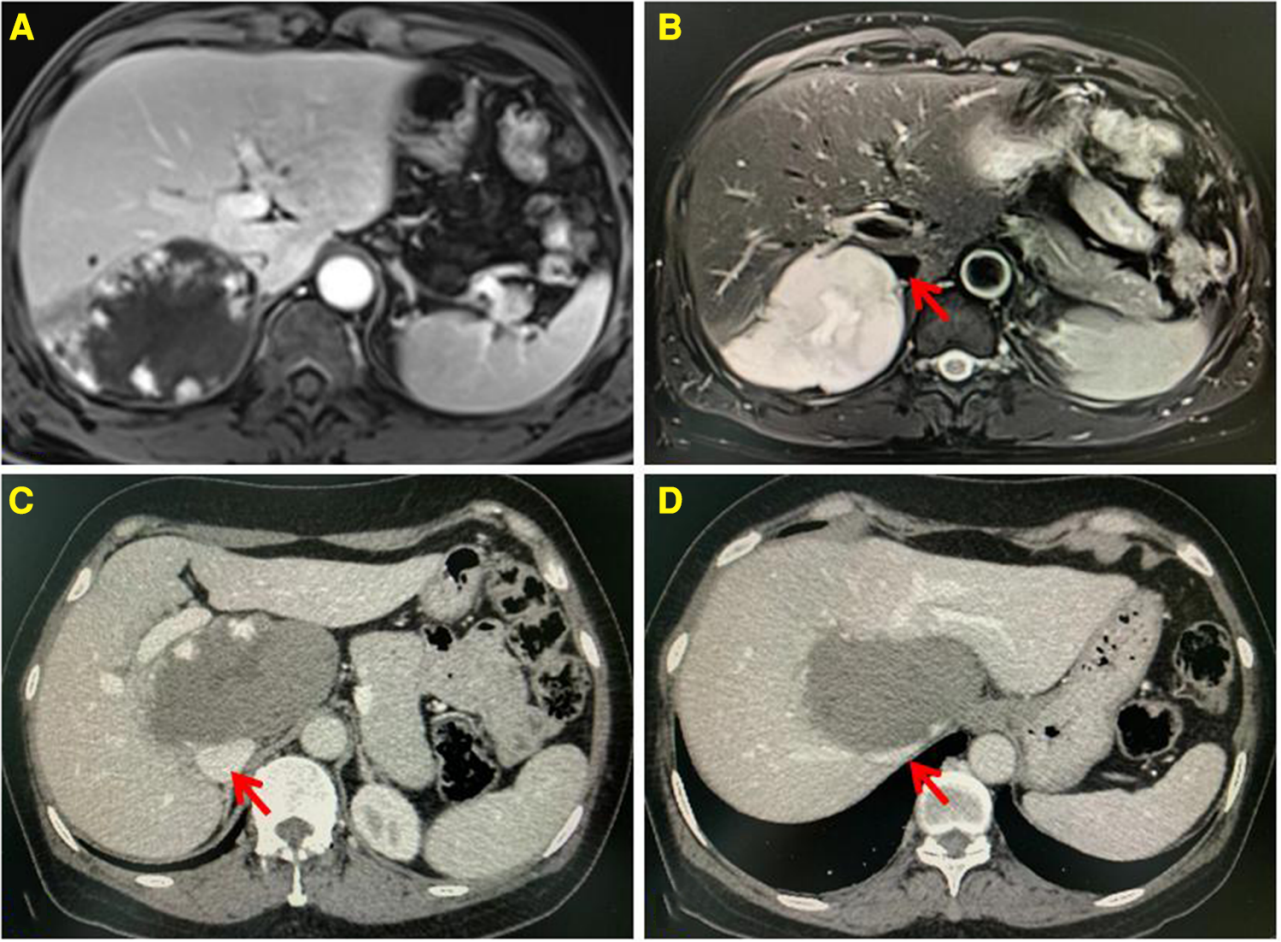
Enhanced MRI showing the lesion located in the segment VII, low signal on T1WI, and high signal on T2WI. Progressive enhancement and high strength mural nodules can be seen on the enhanced scan (**A,B**). Enhanced CT showing the lesion located in the segment I, having a low density. Progressive enhancement can be seen on the enhanced scan (**C,D**). The inferior vena cava is compressed by the lesion. The red arrow indicates the IVC.

Preoperative evaluation included detailed clinical history, radiological investigations, laboratory tests, and ECGs. The location of HH and its relationship with intrahepatic blood vessels or the inferior vena cava were evaluated by enhanced CT or MRI, as shown in Figure 1. Special locations were defined as the tumor located in segments I, IVa, VII, and VIII. HHs located in segments II, III, IVb, V, and VI were defined as the normal location. For patients older than 60 years, echocardiography and pulmonary function examination were necessary to assess the risk of general anesthesia.

## 3. Surgical procedures

### 3.1. Body position and layout of the trocar

Under satisfactory general endotracheal anesthesia, patients were positioned in the reverse Trendelenburg position and their legs were separated. The five-hole method was used routinely. A 10-mm trocar was placed under the umbilical cord as the observation hole. After exploring the specific conditions of the abdominal cavity and hepatic hemangioma, the operating trocar was symmetrically arranged in a U-shape with the hemangioma as the center. If the hemangioma was located in the special segments, we adjusted the 10-mm observation trocar to 3 cm on the right of the umbilicus. The position of the operating trocar should also be adjusted according to patient's body habitus and other factors. The operative table was tilted 30°–40° to the left, and the patient's right arm was raised and fixed on the bracket to facilitate exposure and comfortable operation.

### 3.2. Surgery strategy

The surgery strategies of anatomic hepatectomy or hemangioma enucleation were selected according to the location of the HH. We preferred to perform hemangioma enucleation for most HH cases. However, if the HH was located in the left lateral lobe of the liver (segments II and III), a correspondingly left lateral lobectomy was performed. The liver was first mobilized in varying degrees according to the location of the hemangioma. It is necessary to completely mobilize the right half of the liver to facilitate the exposure of tumor bodies and surgical procedures when the hemangiomas are located in the special segments (I, IVa, VII, VIII). Special care was taken to avoid injuring the right adrenal gland, the inferior vena cava, the short hepatic vein draining into the inferior vena cava, the root of the right hepatic vein, and the hemangioma's capsule to avoid uncontrollable bleeding.

### 3.3. Surgical resection

#### 3.3.1. Hemangioma enucleation

The Pringle maneuver was used to block the first hepatic portal, and the intermittent portal and hepatic arterial blood flow could be occluded if necessary. Finding the boundary of the hemangioma was the key to enucleation surgery. If the hemangioma partially protruded from the surface of the liver, the liver's surface tissue was incised from the edge of the liver with an ultrasonic knife following the boundary between HH and normal liver parenchyma. Then, stretching the hemangioma, an aspirator was used to scrape, suck, push, and peel along the hemangioma wall. Blunt separation was performed. As the enucleation progressed, loose connective tissue, small vessels, and bile ducts were transected with the ultrasonic knife. Large vascular structures and bile ducts were clipped individually with Hem-o-lok clips after fully mobilizing and confirming the shape. Then, the hemangioma was removed completely. For those whose hemangioma was located inside the liver parenchyma, it was difficult for us to accurately locate the edge of the hemangioma and the important vascular structures around the hemangioma on the liver's surface as we were lack of endoscopic ultrasound equipment. We usually mark the pre-resection line on the liver's surface about proximal 2–3 cm from the edge of the extrahepatic tumor body. The liver parenchyma was incised along the pre-resection line with an ultrasonic knife layer by layer, from bottom to top, left to right, and shallow to deep to find the wall of the hemangioma. Then, the aspirator was used to conduct blunt dissection along the boundary between HH and normal liver parenchyma by scraping and aspirating. During this procedure, operation acts should be gentle to avoid tearing blood vessels or damaging the hemangioma wall, which would cause uncontrollable bleeding.

#### 3.3.2. Left lateral lobectomy

The left coronary ligament and left triangular ligament were incised utilizing an ultrasonic knife to incise the liver parenchyma along the left side of the falciform ligament from shallow to deep, front to back, to the surface of the Glisson sheath. Hem-o-lok clips were used to control the bile ducts and vessels. An Endo-GIA autosuture universal stapler was used to transect and close the Glisson sheath and left hepatic vein.

**Figure 2:**
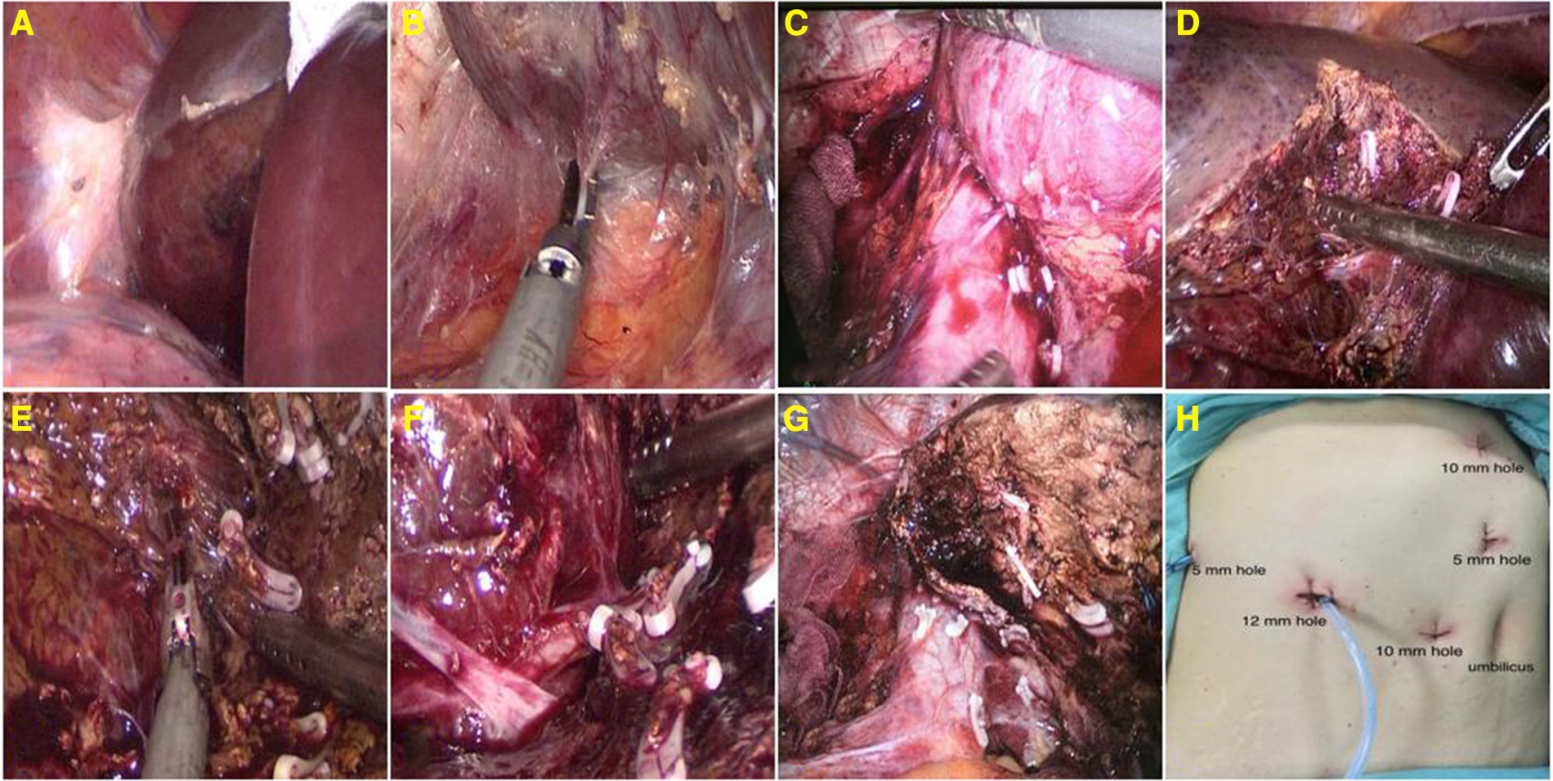
Expose and detach the right triangular ligament (**A**); detach the liver's bare area and retrohepatic space (**B**); segregate and amputate the short hepatic vein at the third hepatic portal (**C**); stripping the hemangioma with aspirator and ultrasonic knife (**D–F**); the liver transection surface (**G**); and layout of trocars (**H**).

Following specimen removal, the liver transection surface was irrigated with normal saline and then carefully inspected for bleeding or biliary leakage. The bleeding sites were stopped by bipolar electrocoagulation or argon spraying or sutured with prolene sutures. Biliary leakage was sutured with prolene sutures. Then, the liver transection surface was irrigated with normal saline repeatedly to ensure no bleeding and biliary leakage. We routinely placed the excised hemangioma into a specimen bag through a 12-mm trocar. Then, the specimen was chopped into pieces and taken out gradually. Two abdominal drainage tubes were placed on the liver transection surface, which were led out from the trocar hole of the abdominal wall and fixed. In the surgical procedure, low central venous pressure was routinely maintained at 0–4 cm H_2_O to reduce bleeding. Meanwhile, the first hepatic portal was blocked by the mode of “15 min + 5 min”; that is, the hepatic portal was blocked for 15 min and then removed for 5 min before the next occlusion. The chief surgical procedures are shown in Figure 2.

### 3.4. Observation index

Perioperative laboratory data were collected along with the location and size of the HH. Surgical approach and operative data including operation time, estimated blood loss, and rate of transfusion were collected. Meanwhile, for postoperative recovery, biochemical indexes, exhaust time, complications, and postoperative hospital stay were collected. According to the location of the HH, patients were divided into two groups including the special location group (SLG) and the normal location group (NLG).

### 3.5. Statistical analysis

All variables were analyzed by SPSS 20.0 software, continuous variables were expressed as means ± SD, and a *t*-test was used to compare the groups. Categorical variables were tested by a *χ*^2^ test and Fisher's accurate test. *p* < 0.05 was considered statistically significant.

## 4. Results

### 4.1. General data

Of the 58 HH patients in this study, 27 patients were enrolled in the special location group and 31 patients in the normal location group. As shown in Table 1, there were no significant differences in age (51.8 ± 4.7 vs. 53.3 ± 5.8 years, *p* = 0.288), gender (*p* = 0.331), body mass index (BMI) (25.3 ± 2.8 vs. 24.4 ± 2.1, *p* = 0.168), the maximum diameter of hemangioma (11.7 ± 2.3 vs. 12.8 ± 5.4 cm, *p* = 0.330), ASA risk grading (*p* = 0.615), and comorbidities (*p* > 0.05) between the two groups.

**Table 1:** General data of patients.

Index	SLG (*n* = 27)	NLG (*n* = 31)	*p*-value
Age (years)	51.8 ± 4.7	53.3 ± 5.8	0.288
Gender			
Male, *n* (%)	8 (29.6)	13 (41.9)	0.331
Female, *n* (%)	19 (70.4)	18 (58.1)	
BMI (kg/m^2^)	25.3 ± 2.8	24.4 ± 2.1	0.168
Diameter (cm)	11.7 ± 2.3	12.8 ± 5.4	0.330
ASA grading			
I, *n* (%)	19 (70.4)	25 (80.6)	0.615
II, *n* (%)	6 (22.2)	5 (16.1)	
III, *n* (%)	2 (7.4)	1 (3.3)	
Comorbidities			
Diabetes mellitus, *n* (%)	4 (14.8)	5 (16.1)	0.822
Hypertension, *n* (%)	6 (22.2)	9 (29.0)	0.554
Cardiovascular disease, *n* (%)	3 (11.1)	3 (9.7)	0.800
Others, *n* (%)	5 (18.5)	4 (12.9)	0.822

SLG, special location group; NLG, normal location group; BMI, body mass index.

### 4.2. Intraoperative outcomes

Twenty-three patients were successfully treated by complete hemangioma enucleation, and four patients were shifted to laparotomy due to the difficulty in control of intraoperative bleeding, with a conversion rate of 14.8% in the SLG. Among them, two cases were caused by an injury to the root of the right hepatic vein during the removal of hemangioma in segment VII, one case was caused by an injury to the hemangioma wall during the removal of hemangioma in segment I, and another case was caused by an injury to the short hepatic vein during the removal of hemangioma in segment I. No case was converted to laparotomy in the NLG. There was no significant difference between the two groups (*p* = 0.089). However, there were significant differences in operation time (258.4 ± 49.2 vs. 186.2 ± 51.8 min, *p* < 0.001), intraoperative blood loss (466.7 ± 235.7 vs. 259.3 ± 92.6 ml, *p* < 0.001), and intraoperative blood transfusion rate (29.6% vs. 6.5%, *p* = 0.047) (Table 2). There was no death case in the two groups.

**Table 2:** Intraoperative outcomes of patients between the two groups.

Index	SLG (*n* = 27)	NLG (*n* = 31)	OR	95% CI	*p*-value
Operation time (min)	258.4 ± 49.2	186.2 ± 51.8	—	45.51–98.89	<0.001
Intraoperative blood loss (ml)	466.7 ± 235.7	259.3 ± 92.6	—	115.48–299.32	<0.001
Intraoperative blood transfusion, *n* (%)	8 (29.6)	2 (6.5)	6.105	1.168–31.916	0.047
Open conversion, *n* (%)	4 (14.8)	0 (0)	—	—	0.089
Mortality, *n* (%)	0 (0)	0 (0)	—	—	1.000

SLG, special location group; NLG, normal location group.

### 4.3. Postoperative outcomes and complications

In the postoperative course, the levels of serum transaminase and bilirubin in the early stage showed an increasing trend, reaching a peak on the third or fourth day after the operation, and then gradually decreased to the normal range. There were no significant differences in biochemical indexes between the two groups (*p* > 0.05). However, the patient's exhaust time and postoperative hospital stay of the SLG were significantly longer than that of the NLG, which were 3.8 ± 1.4 vs. 2.9 ± 0.8 days (*p* = 0.03) and 11.5 ± 3.4 vs. 7.3 ± 2.6 days (*p* < 0.01) respectively. Postoperative complications including pleural effusion (29.6% vs. 19.4%, *p* = 0.362), ascites (11.1% vs. 9.7%, *p* = 0.800), biliary leakage (11.1% vs. 12.9%, *p* = 0.845), and deep vein thrombosis (7.4% vs. 6.5%, *p* = 0.735) were comparable between the two groups (Table 3). There were no perioperative deaths.

**Table 3:** Postoperative outcomes and complications of patients between the two groups.

Index	SLG (*n* = 27)	NLG (*n* = 31)	OR	95% CI	*p*-value
Postoperative data					
ALT (U/L)	382.5 ± 138.3	327.4 ± 148.2	—	—	0.151
AST (U/L)	407.4 ± 158.2	341.1 ± 129.7	—	—	0.085
TBIL (μmmol/L)	77.2 ± 18.7	69.4 ± 21.5	—	—	0.149
ALB (g/L)	35.7 ± 2.6	36.8 ± 3.3	—	—	0.168
Postoperative recovery					
Exhaust time (days)	3.8 ± 1.4	2.9 ± 0.8	—	0.31–1.49	0.003
Postoperative hospital stay (days)	11.5 ± 3.4	7.3 ± 2.6	—	2.62–5.78	<0.001
Complications					
Pleural effusion, *n* (%)	8 (29.6)	6 (19.4)	—	—	0.362
Ascites, *n* (%)	3 (11.1)	3 (9.7)	—	—	0.800
Biliary leakage, *n* (%)	3 (11.1)	4 (12.9)	—	—	0.845
Deep vein thrombosis, *n* (%)	2 (7.4)	2 (6.5)	—	—	0.735
Mortality, *n* (%)	0 (0.0)	0 (0.0)	—	—	1

SLG, special location group; NLG, normal location group; ALT, alanine aminotransferase; AST,aspartate transaminase; TBIL, total bilirubin; ALB, albumin.

The levels of biochemical indexes in Table 3 were tested on the third day after the operation.

## 5. Discussion

A hepatic hemangioma can generally be observed and requires intervention only if it is symptomatic. Surgical resection is the most effective and widely accepted treatment. Over the past few decades, the laparoscopic liver approach has gained widespread acceptance and become the first choice for the treatment of HH, parallel with the continued evolution of surgical expertise and improved instrumentation. Its safety, efficacy, and advantages have been effectively verified in clinical practice. Relevant clinical studies showed that laparoscopic surgery for HH has the advantages of less intraoperative bleeding, lower postoperative complication rate, faster recovery, and shorter hospital stay than traditional open surgery (27–30). These advantages are mainly attributed to the clear and magnifying vision of the operational field in laparoscopy and the application of laparoscopic energy instruments such as ultrasound scalpels, bipolar electrocoagulation, and argon spraying, which are beneficial to hemostasis.

In terms of the selection of surgical methods, the selected surgical procedures are different according to the size and location of HH. An extremely giant HH of more than 20 cm is typically resected using the open approach (31). As the extremely giant HH occupies most of the intraperitoneal space, leading to limited operating space, it will increase the risk of intraoperative massive hemorrhage and mortality. Laparoscopic left lateral lobectomy has become the standard operation method for patients with hemangiomas in segments II and III (32). For the extrahepatic hemangioma of other liver segments, laparoscopic hemangioma enucleation is preferred. As the hepatic hemangioma grows expansively, it squeezes the surrounding normal liver parenchyma in the process of growth and forms a layer of a fibrous capsule around it. There is a boundary between the fibrous capsule and the normal liver parenchyma. Stripping the hemangioma along this boundary is conducive to retaining more normal liver parenchyma and reducing the incidence of postoperative complications such as bleeding and biliary fistula (33, 34). It was once considered a prohibited area of surgery for patients with HH located in the special hepatic segments (I, IVa, VII, VIII), which had bad visualization and were adjacent to important vessels such as hepatic veins and the inferior vena cava, associating the surgery of HHs in this region to characteristics of “more intraoperative bleeding, more postoperative complications, and high mortality.” With the continued evolution of surgical expertise and improved instrumentation, laparoscopic enucleation of hemangiomas located in the right posterior lobe or caudate lobe of the liver has been carried out and has gradually become a routine operation. However, it is still a big challenge for surgeons and has an extremely high requirement for the tacit cooperation of the team. In this study, the operation time, intraoperative blood loss, and intraoperative blood transfusion rate of the SLG were significantly higher than those of the NLG (*p* < 0.05). In the postoperative course, the levels of serum transaminase and bilirubin in the early stage showed an increasing trend, reaching a peak on the third or fourth day after the operation, and then gradually decreased to the normal range with the medical therapy. There were no significant differences in biochemical indexes and postoperative complications between the two groups (*p* > 0.05). However, the patient's exhaust time and postoperative hospital stay of the SLG were significantly longer than that of the NLG. Also, there were no perioperative deaths in the two groups. This result demonstrated that hemangiomas located in special liver segments have a higher risk of intraoperative hemorrhage, but a timely shift to laparotomy can ensure the safety of patients and postoperative recovery.

Based on this study, we have summarized some experiences. First, the fixed combination and skilled operation team are the basis for the successful implementation of complex laparoscopic surgery, especially in the event of severe bleeding and other critical situations during the operation. Mutual trust and effective cooperation between teams are extremely important. Second, it is an important prerequisite to fully expose the tumor body. The liver is an organ rich in blood supply, and its internal pipeline structure is complex. Bleeding often occurs due to blood vessel damage during surgery. The special locations' narrow space and bad visualization make the laparoscopic suture difficult and increase the rate of laparotomy and the risk of death. Therefore, completely freeing the right half of the liver to facilitate exposure is extremely important for the surgical operation and hemostasis. Third, finding and following the boundary between the hemangioma and normal liver parenchyma is the key to enucleation and avoiding hemorrhage. In addition, intermittent blocking of the first hepatic portal can not only reduce hemorrhage during the operation but also can reduce the volume of blood within the HH, facilitating its identification and dissection. Fourth, we should keep a stable attitude and gentle action. The hepatic hemangioma's tissue is relatively soft, especially the cavernous hemangioma. Mechanical touching, pulling, and other operations easily damage the tumor's capsule and lead to bleeding. Therefore, selecting appropriate operating instruments for traction is necessary, and the strength should be kept moderate to avoid membrane tears caused by excessive traction. An aspirator is a good “weapon” for stripping the hemangioma. The aspirator has a round and blunt front end. It is not easy to damage the hemangioma wall and the venous system compressed by the hemangioma when stripping the hemangioma with an aspirator. Meanwhile, keeping the aspirator with tiny suction intermittently helps to maintain a clear vision of the operation area, facilitates the exposure of important vascular structures around the tumor body, and avoids accidental injury and massive bleeding. It is advised not to cut off the vascular structures in a hurry without fully exposing their shapes. This helps us to avoid damaging important blood vessels by mistake. Moreover, this is especially important when a giant hemangioma compresses hepatic veins and other important blood vessels. Fifth, strong psychological quality and prompt decision to a shift to laparotomy assure to maintain the safety of surgery when encountering uncontrollable bleeding.

The present study has several limitations. First, the sample size of the overall study is relatively small. This is the clinical reality of a developing medical center and a single-center study. Second, we lack advanced medical equipment, such as endoscopic ultrasound and cavitation ultrasonic surgical aspirator, and the like. This may have influenced the outcomes. Third, we are in the early stage of such surgery, and the learning curve effect may have influenced the outcomes. A well-designed multicenter study with a large sample size would be ideal in the future.

## 6. Conclusion

In conclusion, laparoscopic enucleation of hemangioma in special hepatic segments is difficult and has a critical risk of massive bleeding during surgery. Meanwhile, it is also safe, feasible, and effective. Grasping indications of laparoscopic surgery, good preoperative planning, skilled laparoscopic technique, and tacit teamwork are significant factors for the success of this surgery.

## Data Availability

The raw data supporting the conclusions of this article will be made available by the authors without undue reservation.
